# Identifying a molecular profile to predict the risk of recurrence in high‐intermediate risk endometrial cancer

**DOI:** 10.1002/cam4.4247

**Published:** 2021-11-02

**Authors:** Rebecca C. Arend, Carly B. Scalise, Jhalak Dholakia, Maahum Z. Kamal, Haley B. Thigpen, David Crossman, Warner K. Huh, Charles A. Leath

**Affiliations:** ^1^ Division of Gynecologic Oncology, Department of Obstetrics and Gynecology University of Alabama at Birmingham Birmingham Alabama USA; ^2^ University of Alabama at Birmingham School of Medicine Birmingham Alabama USA; ^3^ Department of Genetics University of Alabama at Birmingham Birmingham Alabama USA

**Keywords:** biomarker, endometrial cancer, high‐intermediate risk, mutations, recurrence, survival

## Abstract

**Background:**

Patients with high‐intermediate risk endometrial cancer (H‐IR EMCA) have an elevated risk of recurrence compared to low‐risk counterparts. Many H‐IR EMCA patients are treated with radiation or chemotherapy, but their overall survival is not significantly impacted by treatment. The objective of this study was to compare molecular profiles of H‐IR EMCA patients with disease recurrence to those without to identify characteristics that could better predict patient outcomes.

**Methods:**

Tissue was acquired from H‐IR EMCA patients with disease recurrence (n=15) and without disease recurrence (n=15) who had not received adjuvant therapy and performed DNA and RNA analyses.

**Results:**

In recurrent population, 5 patients had matchingrecurrent and initial tumor tissues. Of note, 5/7 (71%) African Americanpatients had disease recurrence compared to 10/23 (43%) White patients. Inaddition, several new mutations were found in individual patient’s recurrentcompared to initial tumors.

**Conclusions:**

Currently the treatment ofendometrial cancer is rapidly changing with molecular profiling becoming partof the standard of care. Additionally, it and is being incorporated intoclinical trials in this group of patients. The specific gene mutations and RNAexpression signatures that were observed in our small cohort need to bevalidated in larger cohorts to determine their impact.

## Graphical abstract

In gynecologic oncology, endometrial cancer (EMCA) patients considered high‐intermediate risk (H‐IR) have elevated recurrence compared to low‐risk patients. In this study, we compared changes in DNA and RNA between 15 H‐IR patients with disease recurrence to 15 H‐IR patients without disease recurrence. This comparison allowed for us to highlight the specific players that contributed to a patient's disease recurrence, and in doing so, expose vulnerable parts of the cancer that we could use to better predict patient treatment strategies and outcomes.

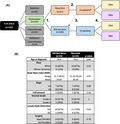

## 1 INTRODUCTION

Endometrial adenocarcinoma (EMCA) is the most common cancer of the female reproductive tract in the United States.^1^ Approximately 65,620 women will be diagnosed in 2020, resulting in 12,590 deaths.^2^ EMCA incidence rates are rising in concordance with increasing obesity rates and shifts in reproductive trends.^3^ Broadly, patients with EMCA have a more favorable prognosis compared to women with other gynecologic malignancies.^4^ Over 67% of women are diagnosed with uterus‐confined disease,^4^ and standard of care surgical management via hysterectomy with bilateral salpingo‐oophorectomy is usually curative.^5^ However, a *subset of patients* with early disease is at *increased risk for disease recurrence*.^6^

Patients are classified as either low, high, or high‐intermediate risk (H‐IR) based on the presence or absence of specific clinical and pathologic criteria associated with worsened prognosis. The Proactive Molecular Risk Classifier for Endometrial Cancer (ProMisE) molecular classification system can use the protein expression of p53, MMR proteins (PMS2 and MSH6), and POLE exonuclease domain to predict poor, intermediate, and improved patient outcomes, respectively.^7^ We used *GOG definition, not PORTEC, of H‐IR EMCA* including deep myometrial invasion, grade 2 or 3 histology, lymphovascular space invasion (LVSI), and patient age. The recurrence rate in H‐IR EMCA patients is elevated compared to their low‐risk counterparts (20% and 2.6%, respectively), but the role of adjuvant therapy is controversial.^8^ The majority of patients will recur locally and can undergo salvage treatment; those with distant recurrences have few effective treatment options and a poorer prognosis.^9^ Per GOG‐99, adjuvant radiotherapy (RT) significantly decreased the recurrence in H‐IR patients but did not impact the overall survival. Additionally, RT carries its own risks of adverse short‐ and long‐term toxicities.^10^ This highlights the need to better stratify H‐IR EMCA patients in order to identify those who would most benefit from adjuvant therapy while minimizing treatment to those at lower risk.

The use of histological characterization for risk stratification offers insight into the pathological mechanisms influencing the likelihood of HI‐R EMCA recurrence. Unfortunately, there is often inter‐observer discrepancy when classifying subtypes, indicating the need for a less subjective classification system.^11^ Advances in molecular analysis have identified key mechanisms in EMCA pathogenesis and progression, as well as potential therapeutic targets.^12^ Studying these tumors on a molecular level also allows additional classification regarding recurrence risk.^13^ For example, *PTEN* is frequently mutated in endometrioid EMCA, but does not cause disease on its own.^13^ However, alterations in *PTEN* and *ARID1A* might synergistically predispose women to atypical hyperplasia/endometrioid intraepithelial neoplasia, an EMCA precursor lesion.^14^ Rather than focusing on individual genes, Wang et al. developed and validated a six gene signature including *CTSW*, *PCSK4*, *LRRC8D*, *TNFRSF18*, *IHH*, and *CDK2NA* to predict EMCA prognosis.^15^ Other studies used the four well‐known The Cancer Genome Atlas (TCGA) molecular subclasses (POLE ultra‐mutated, microsatellite unstable, copy number low, and copy number high) to risk stratify patients.^16^ These studies all experience one significant limitation and potential confounder in assessing recurrence and survival: patients in these cohorts were not controlled for receipt of adjuvant treatment. Additionally, the feasibility of performing whole exome sequencing on all patients is not practical. Therefore, additional research is needed to identify and/or validate genes and molecular pathways contributing to H‐IR EMCA recurrence using Clinical Laboratory Improvement Amendments (CLIA)‐ and/or Food and Drug Administration (FDA)‐approved diagnostic testing. The identification of components influencing disease recurrence could decrease overtreatment and contribute to improved treatment stratification of H‐IR EMCA patients.^12^ This could enable physicians to better assess recurrence risk and limit adjuvant treatment to patients at highest risk, limiting unnecessary therapy and its associated adverse outcomes and healthcare costs. The objective of this study was to molecularly profile primary tumors from patients with H‐IR EMCA who did not receive adjuvant treatment and experienced recurrence to a matched cohort of patients without recurrence. Our goal was to demonstrate quantifiable molecular differences, at the time of original diagnosis, between tumors from patients who do and do not experience recurrence, to better characterize predictive molecular risk factors for recurrence.

## 2 MATERIALS AND METHODS

### 2.1 Chart review

Under an Institutional Review Board‐approved protocol at the University of Alabama at Birmingham (UAB), all patients with biopsy‐proven EMCA who underwent surgery between 2000 and 2010 at UAB and met criteria for H‐IR EMCA disease based on GOG‐99 criteria (although outer 1/3 was replaced by outer 1/2) were reviewed. Of the 292 patients that met H‐IR EMCA criteria, 222 were observed (without adjuvant treatment). Of those treated, 44 received adjuvant RT, 21 received adjuvant chemotherapy, and 4 received both. Clinical data were collected on all patients; molecular analysis was performed on a subset (*n* = 30) of patients who were observed.

### 2.2 Tumor material

Formalin‐fixed paraffin‐embedded (FFPE) slides were made from archival tissue (original hysterectomy specimen) from 15 patients who recurred and 15 patients who did not. In the subset of those who were observed after surgery without recurrence, tissue was available for 15 patients. These patients formed the “control” group. These were matched to 15 patients with disease recurrence based on recurrence, race, and grade. In addition, 5 of 15 patients who recurred had tissue available from their recurrence. In these patients, FFPE slides were made from both primary and recurrent tumors.

### 2.3 DNA analysis

Tumor DNA was isolated by manual microdissection followed by NextSeq using CARIS’s custom‐designed SureSelect XT assay of 592 whole‐gene targets (point mutations, copy number variations, and insertions/deletions), 53 RNA gene fusions, microsatellite instability, and total mutational load was performed on the above‐defined 35 archival FFPE tumors. All variants were detected with >99% confidence based on allele frequency and average coverage of >500 and an analytic sensitivity of 5%. Genetic variants identified were interpreted by board‐certified molecular geneticists and categorized based on American College of Medical Genetics standards.

Differences in the number of genes with DNA mutations between recurrent (*n* = 15) and non‐recurrent (*n* = 15) patient primary tumors were assessed using a two‐tailed nonparametric Mann–Whitney *U* test. Differences in the number of mutated genes between primary and recurrent tumor samples from the same patient (*n* = 5) were evaluated using a one‐tailed nonparametric Wilcoxon matched‐pairs signed rank test. DNA mutation analyses were performed using GraphPad Prism version 8.4.2 for Windows (GraphPad Software; www.graphpad.com) with *α* = 0.05. Odds ratios and 95% CIs were calculated then verified using on online odds ratio calculator tool (https://select‐statistics.co.uk/calculators/confidence‐interval‐calculator‐odds‐ratio/).

### 2.4 RNA analysis

Tumor RNA was isolated from FFPE blocks. Gene expression data were collected for 770 genes using the Nanostring nCounter^®^ PanCancer Pathways Panel on the same samples as above in DNA analysis. Molecular profiles and pathway analysis of the cohorts (recurrence vs. no recurrence; primary vs. recurrent) were compared using nSolver Advanced Analysis Software^®^ and Ingenuity Pathways Analysis (Ingenuity^®^ Systems; www.ingenuity.com). Genes were evaluated using a fold change of ±1.5 and a *p* value of <0.05. For generating networks, a dataset containing gene identifiers and corresponding expression values was uploaded into Ingenuity Pathways Analysis software. Each identifier was mapped to its corresponding object in Ingenuity's Knowledge Base. A fold change cutoff of ±1.5 was set to identify molecules whose expression was significantly differentially regulated. These molecules, called network eligible molecules, were overlaid onto a global molecular network developed from information contained in Ingenuity's Knowledge Base. Networks of network eligible molecules were then algorithmically generated based on their connectivity. The Functional Analysis Tool identified the biological functions and/or diseases that were most significant to the entire dataset. Molecules from the dataset that met the fold change cutoff of ±1.5 and were associated with biological functions and/or diseases in Ingenuity's Knowledge Base were considered for the analysis. Right‐tailed Fisher's exact test was used to calculate a *p* value determining the probability that each biological function and/or disease assigned to that dataset is due to chance alone.^17^

### 2.5 Survival analysis

cBioPortal for Cancer Genomics (https://www.cbioportal.org) was used to produce Kaplan–Meir curves and copy number alteration (CNA) frequency % bar graphs from the Uterine Corpus Endometrial Carcinoma (TCGA; PanCancer Atlas) dataset (*n* = 529).

### 2.6 CADD analysis

Variants from the DNA alterations identified in the DNA analysis were uploaded to the combined annotation‐dependent depletion (CADD) website (https://cadd.gs.washington.edu/score) using GRCh37‐v1.4 for CADD scoring.^17^ The gene of any variants with either a CADD score between 20 and 30, or ≥30 was then matched with any upstream regulator gene ingenuity found in the NanoString expression data.

## 3 RESULTS

### 3.1 DNA analysis highlights mutation profiles that correlate with recurrence and survival in H‐IR EMCA patient samples

**FIGURE 1:**
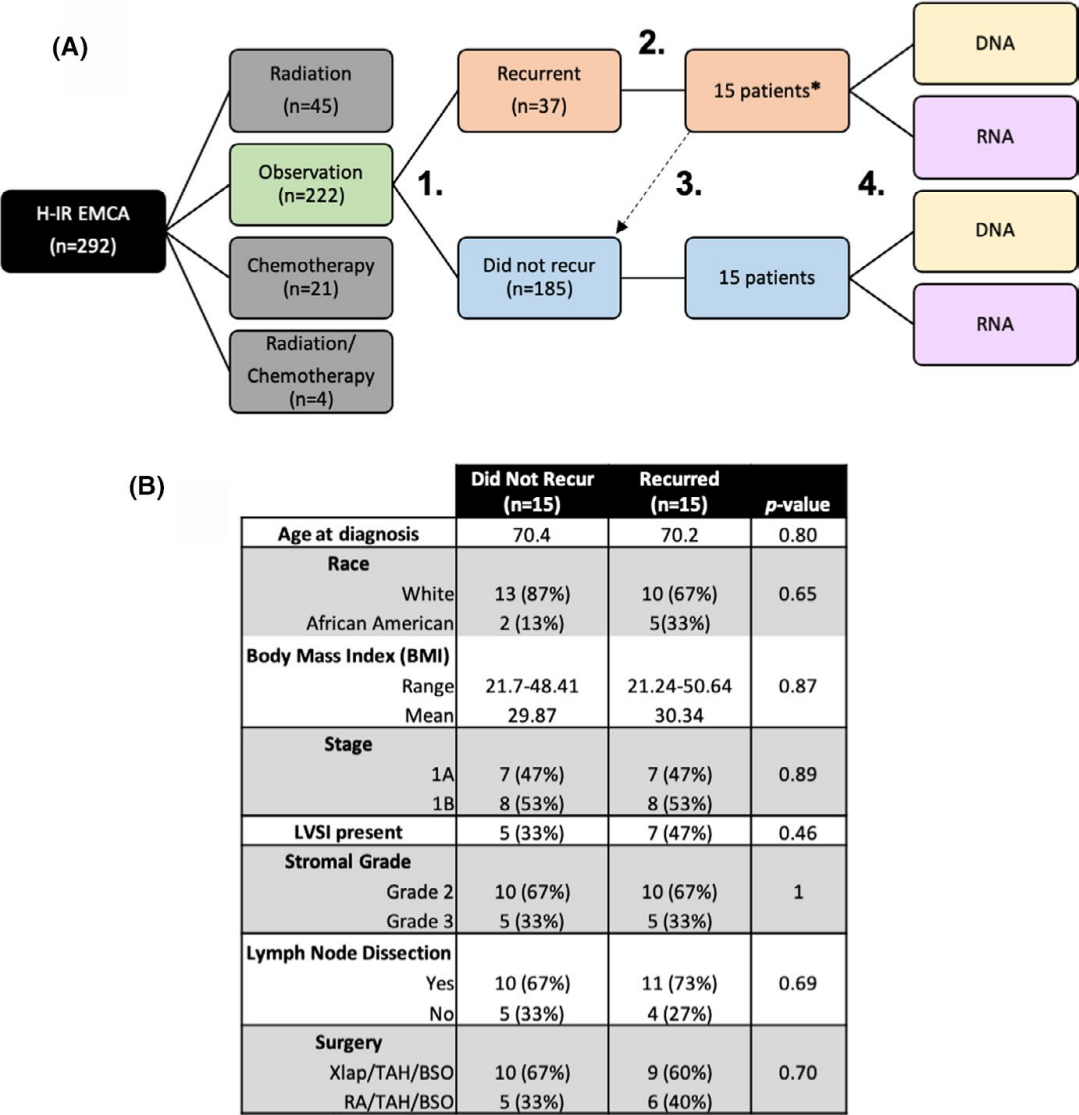
Flow chart of study design. (A) Between 2000 and 2010 patients that met H‐IR disease based on Gynecologic Oncology Group‐99 were reviewed. Of the 292 patients that met H‐IR criteria, 44 patients received adjuvant radiotherapy, 21 received adjuvant therapy, 4 were treated with both radiotherapy and chemotherapy, and 222 did not receive adjuvant treatment (observation). From the 222 patients that did not receive adjuvant therapy, (1.) 185 did not have disease recurrence and 37 did have disease recurrence. (2.) From the patients with disease recurrence, 15 patients were matched based on recurrence, race, and grade (3.) to patients that did not have disease recurrence. (4.) DNA and RNA analyses were performed on these 30 patient samples. *From the 15 patients that had disease recurrence, 5 patients had both their primary and recurrent tumors analyzed. (B) Patient demographics and tumor characteristics. H‐IR EMCA, high‐intermediate risk endometrial cancer; LVSI, lymphovascular space invasion

**FIGURE 2:**
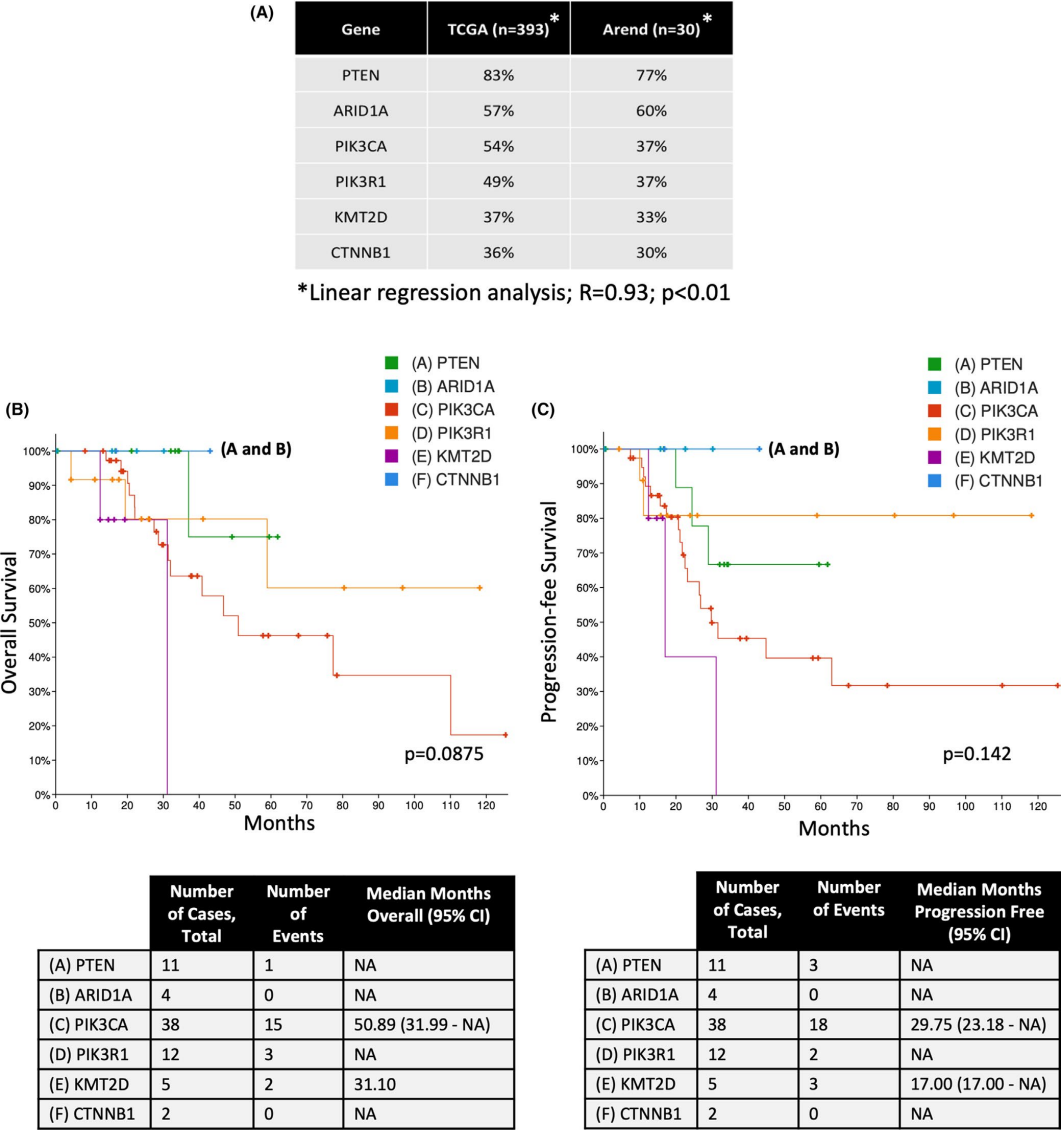
F 2 DNA analysis highlights the gene expression profiles that correlate with survival in H‐IR EMCA patients. Next‐generation sequencing using a 592 DNA panel was used to analyze the most frequently mutated genes in 30 primary tumor samples (Arend dataset) from 15 H‐IR EMCA patients with disease recurrence and 15 H‐IR EMCA that did not have disease recurrence matched on age, grade, LVSI, race, and disease stage. (A) Percentages of patients with gene mutations present at primary diagnosis of endometrial adenocarcinoma TCGA (*n* = 393) and Arend (*n* = 30) datasets. Uterine Corpus Endometrial Carcinoma (TCGA, PanCancer Atlas) dataset (*n* = 529) from cBioPortal for Cancer Genomics illustrating the effects of each gene mutation on (B) overall survival (OS) with total number of cases and events and median months of OS, and (C) progression‐free survival (PFS) with total number of cases and events and median months of PFS. H‐IR EMCA, high‐intermediate risk endometrial cancer; LVSI, lymphovascular space invasion

Next‐generation sequencing (NGS) using a 592 DNA panel was used to analyze the most frequently mutated genes in 15 primary tumor samples from HI‐R EMCA patients with disease recurrence compared to 15 primary tumor samples from H‐IR EMCA patients with no disease recurrence matched by recurrence, race, and grade (Figure 1A,B). Among the top 10 most frequently mutated genes in primary tumors from both the Arend (30 patients) and TCGA (393 patients) cohorts were PTEN, ARID1A, PIK3CA, PIK3R1, KMT2D, and CTNNB1 (Figure 2A). Linear regression analysis revealed a strong, significant (*R* = 0.93; *p* = 0.007) relationship between these gene mutation frequencies in the Arend and TCGA cohorts. The effects of each of these gene mutations on patient overall and progression‐free survival (OS and PFS, respectively), from TCGA Uterine Corpus Endometrial Carcinoma dataset (TCGA_Endo) (*n* = 529) are shown in Figure 2B,C.

**FIGURE 3:**
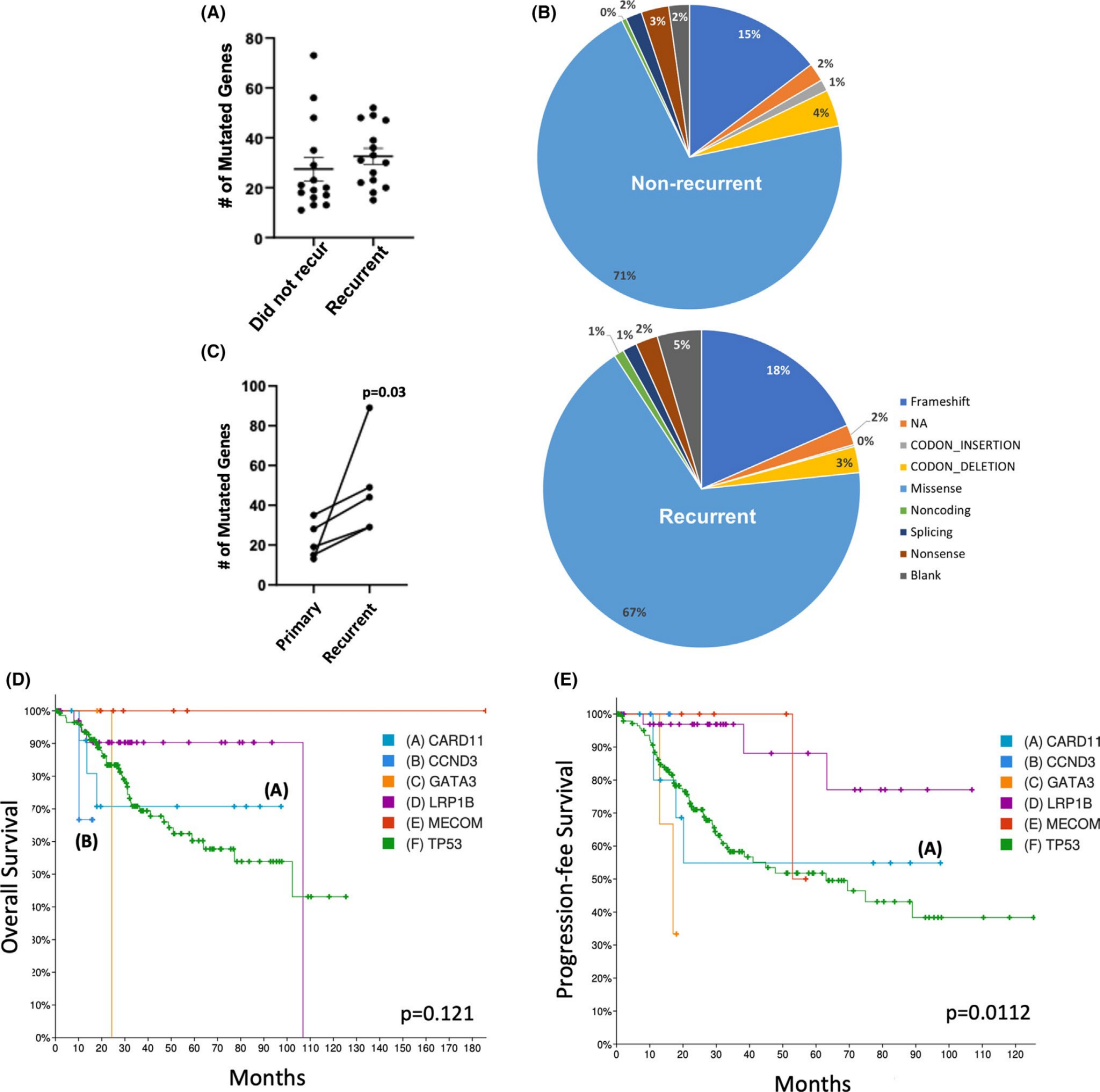
3 DNA analysis highlights the gene expression profiles that correlate with recurrence and survival in H‐IR EMCA patients. In our dataset (Arend *n* = 30) the (A) number of mutations in H‐IR EMCA patient tumors that did not recur and recurrent patient tumors. (B) Types of mutations (e.g., frameshift, codon insertion, codon deletion, missense, noncoding, splicing, and nonsense) seen, with no differences were observed between patients with or without disease recurrence. (C) Number of mutations in individual H‐IR EMCA patient's primary and recurrent tumor (one primary and one recurrent tumor sample per patient). Individual gene mutations of TP53, LRP1B, CARD11, CCND3, MECOM, and GATA3 in Uterine Corpus Endometrial Carcinoma (TCGA, PanCancer Atlas) dataset (*n* = 529) and their effects on patient (D) overall survival and (E) progression‐free survival. H‐IR EMCA, high‐intermediate risk endometrial cancer

When analyzing the number (Figure 3A; *p* = 0.1040) and types of mutations (e.g., frameshift, codon insertion, codon deletion, missense, noncoding, splicing, and nonsense) (Figure 3B) present in H‐IR EMCA patient tumor samples (Arend cohort), no significant differences were observed between patients with or without disease recurrence. In addition, the same analysis was used to compare changes in gene mutations from the individual patient's primary and recurrent tumors (five patients with both a primary and recurrent tumor sample). In this comparison, the number of mutations were significantly higher (Figure 3C; *p* = 0.0313) in the recurrent compared to primary tumor from the individual patient. To determine if gene mutations present in an individual's primary tumor were conserved in their recurrent tumors, we calculated the percentage of mutations that overlapped between the two groups. Only 30% of gene mutations in the individual patient's primary tumors were present in their recurrent tumors. The top five mutations present in patient recurrent versus primary tumors were TP53, LRP1B, CARD11, CCND3, GATA3, and MECOM. This could suggest that H‐IR EMCA patients harboring these mutations at diagnosis are at an increased risk for recurrence, however further studies are needed to validate. Figure 3D,E shows individual gene mutations and their effects on patient OS and PFS, respectively, in TCGA_Endo dataset.

Next‐generation sequencing identified 22 candidate gene mutations in primary tumors in both the recurrent and non‐recurrent H‐IR EMCA tumors. Odds ratios and 95% CIs were calculated to determine the likelihood of these gene mutations being associated with tumor recurrence. Of these mutations, 13 (including *JAK1*, *SPEN*, *BRD3*, *RNF213*, and *TPR*) demonstrated odds ratios >1 (Figure S1A); although given the limited numbers in this study, all CIs crossed one. JAK1 and SPEN were the top two gene mutations in primary tumors associated with tumor recurrence [OR = 7, 95% CI: 0.71, 69.49] (Figure S1A). Figure S1B,C shows individual gene mutations (JAK1, SPEN, BRD3, RNF213, and TPR) and their effects on patient OS and PFS, respectively, in TCGA_Endo dataset. Given the number of patients, it is difficult to draw definitive conclusions on the significance of these genes given the large CIs.

### 3.2 RNA analysis highlights the gene expression profiles that correlate with recurrence and survival in H‐IR EMCA patients

**FIGURE 4:**
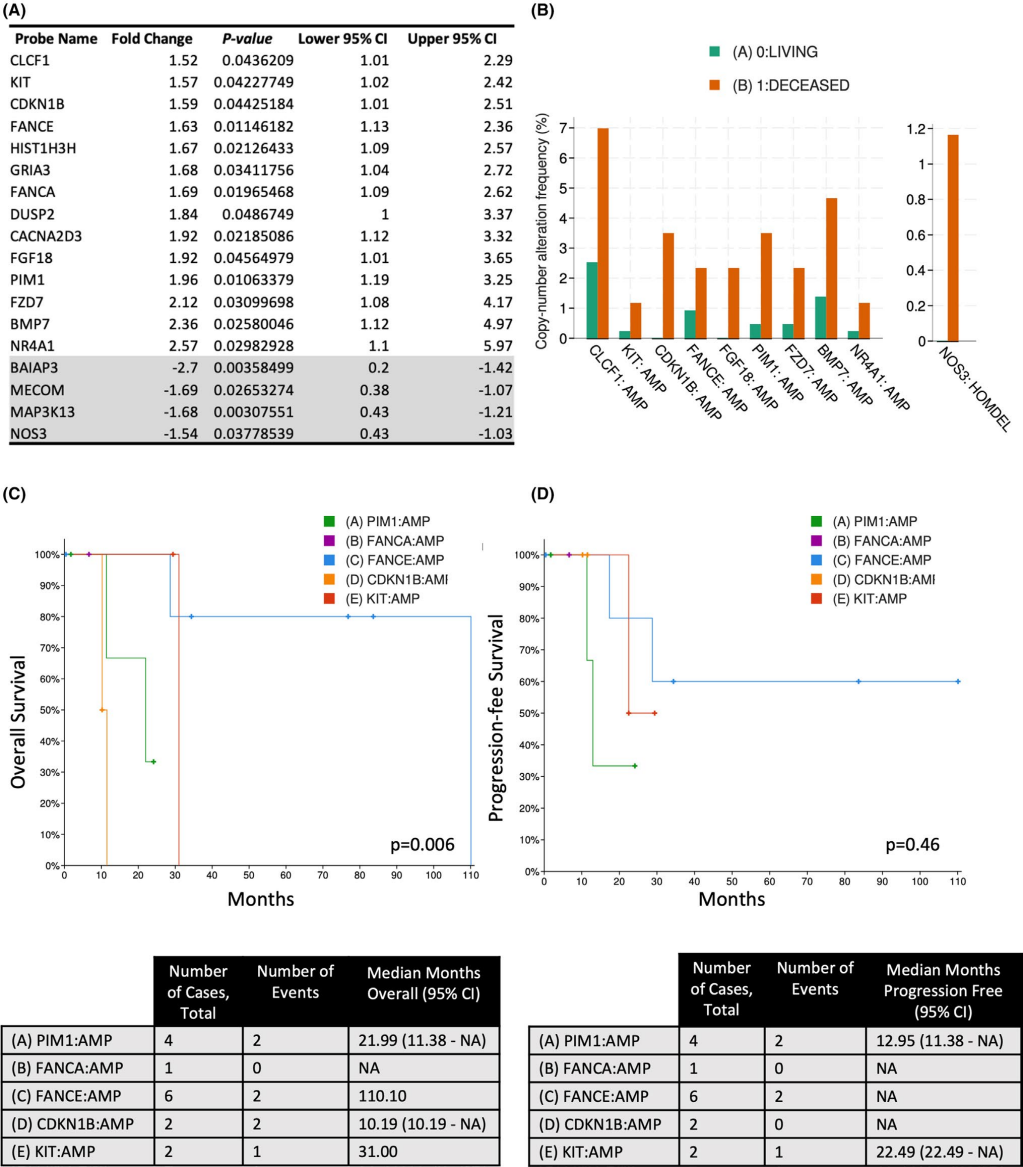
Gene profile associated with H‐IR EMCA recurrence. (A) Our findings revealed 14 genes (CLCF1, KIT, CDKN1B, FANCE, HIST1H3H, GRIA3, FANCA, DUSP2, CACNA2D3, FGF18, PIM1, FZD7, BMP7, and NR4A1) that had >1.5‐fold (range 1.52–2.57) increase in gene expression in H‐IR EMCA patients with disease recurrence versus patients with no disease recurrence. (B) Uterine Corpus Endometrial Carcinoma (TCGA, PanCancer Atlas) dataset (*n* = 529) from cBioPortal for Cancer Genomics illustrating the frequency of copy number alterations of amplified (AMP) or homozygous deleted genes between and their association with patient morbidity (green/orange). Uterine Corpus Endometrial Carcinoma (TCGA, PanCancer Atlas) dataset (*n* = 529) from cBioPortal for Cancer Genomics illustrating the effects of gene expression on (C) OS with total number of cases and events and median months of OS, and (D) PFS with total number of cases and events and median months of PFS. H‐IR EMCA, high‐intermediate risk endometrial cancer; OS, overall survival; PFS, progression‐free survival

Gene expression data were collected for 770 genes on the 35 archival FFPE tumors, and molecular profiles and pathway analyses of the cohorts (recurrent vs. non‐recurrent; primary vs. recurrent) were performed. Our findings revealed 14 genes (CLCF1, KIT, CDKN1B, FANCE, HIST1H3H, GRIA3, FANCA, DUSP2, CACNA2D3, FGF18, PIM1, FZD7, BMP7, and NR4A1) that had >1.5‐fold (range 1.52–2.57) *increase in gene expression* in H‐IR EMCA patients with disease recurrence compared to patients with no disease recurrence (Figure 4A––top/white). Additionally, there were four genes (BAIAO3, MECOM, MAP3K13, and NOS) with >1.5‐fold (range −1.54 to −2.7) *decrease in gene expression* in H‐IR EMCA patients with disease recurrence versus patients with no disease recurrence (Figure 4A––bottom/gray). Elevated CNAs of amplified (AMP) genes (CLCF1, KIT, CDKN1B, FANCE, FGF18, PIM1, FZD7, BMP7, and NR4A1) and homozygous deletion (HOMDEL) of gene, NOS3, were associated with increased patient morbidity (Figure 4B––green/orange). Of the genes that were increased in tumors from patients with disease recurrence versus patients with no recurrence, the effects of their amplification on patient OS and PFS from TCGA_Endo dataset are shown in Figure 4C,D. When comparing genetic information between an individual patient's primary and recurrent tumors, there were 32 genes (top 5: COMP, FGFR3, COL11A1, LEFTY1, and IL1A) that had >1.5‐fold (range 1.51–2.56) *increase in gene expression* in the recurrent versus primary tumors. There were 47 genes (top 5: SFRP4, LEFTY2, HOXA11, ZBTB16, and FOS) with >1.5‐ fold (range −1.65 to −9.57) *decrease in gene expression* in the recurrent versus primary tumors.

Furthermore, 5/7 (71.4%) Black patients had disease recurrence versus 10/23 (43.5%) White patients (Figure 1B). When comparing gene expression changes in Black recurrent (*n* = 5) to White recurrent (*n* = 10) tumors, the top 15 genes that had increased expression included SOST, HMGA2, DKK4, IL10, FST, FGF3, ETV4, FN1, FGF14, HOXA9, MET, CCNE1, SPP1, GNG4, and ITGA8 (Figure S2A––top/white). Within the TCGA_Endo dataset, Black patients harbored elevated CNAs of these AMP genes (SOST, HMGA2, IL10, FST, ETV4, FGF14, HOXA9, SPP1, GNG4, and CCNE1) compared to White counterparts (Figure S2B––red/blue). Furthermore, elevated CNAs of these AMP genes (DKK4, IL10, FGF14, CCNE1, and GNG4) were associated with increased patient morbidity (Figure S2B––green/orange). The top 15 genes that had decreased expression in Black recurrent compared to White recurrent patients included IL1R2, MAPK8IP2, GNG7, BIRC3, GADD45B, KLF4, DKK1, CACNA2D3, CACNA1H, AMH, FGF10, FOXL2, IL8, MMP9, AND IL19 (Figure S2A–bottom/gray). Within TCGA_Endo dataset, Black patients harbored elevated CNAs of HOMDEL genes (MAPK8IP2, GNG7, BIRC3, CACNA2D3, and AMH) compared to White counterparts (Figure S2B––red/blue). Furthermore, elevated CNAs of HOMDEL genes (MAPK8IP2, GNG7, GADD45B, and AMH) were associated with increased patient morbidity (Figure S2C––green/orange). Figure S2D shows the effects of AMP genes, CCNE1 and HOXA9, and HOMDEL genes, GADD45B and BIRC3, on PFS of patients in TCGA_Endo dataset.

Lastly, we compared molecular profiles of Black patients with disease recurrence (*n* = 5) versus Black patients without disease recurrence (*n* = 2). The top 15 genes that had increased expression included DKK4, HMGA2, SOST, ZIC2, NR4A1, NR4A3, BMP7, SFRP2, NKD1, FN1, IL10, FGF19, BMP4, SPP1, and FGF3 (Figure S3A––top/white). Elevated CNAs of these AMP genes (DKK4, BMP7, and BMP4) were associated with increased patient morbidity (Figure S3B). In addition, the top 15 genes that had decreased expression in Black recurrent compared to non‐recurrent patients included AKT1, FGF12, THEM4, SOX17, PLCB1, PLCB4, MYB, FZD8, NOS3, IDH1, MAP3K13, IL5RA, BAIAP3, SIX1, and GPC4 (Figure S3A––bottom/gray). Elevated CNA of HOMDEL of NOS3 was also associated with increased patient morbidity (Figure S3C).

### 3.3 Ingenuity analysis identified multiple pathways and genes associated with recurrence in H‐IR EMCA patients

**FIGURE 5:**
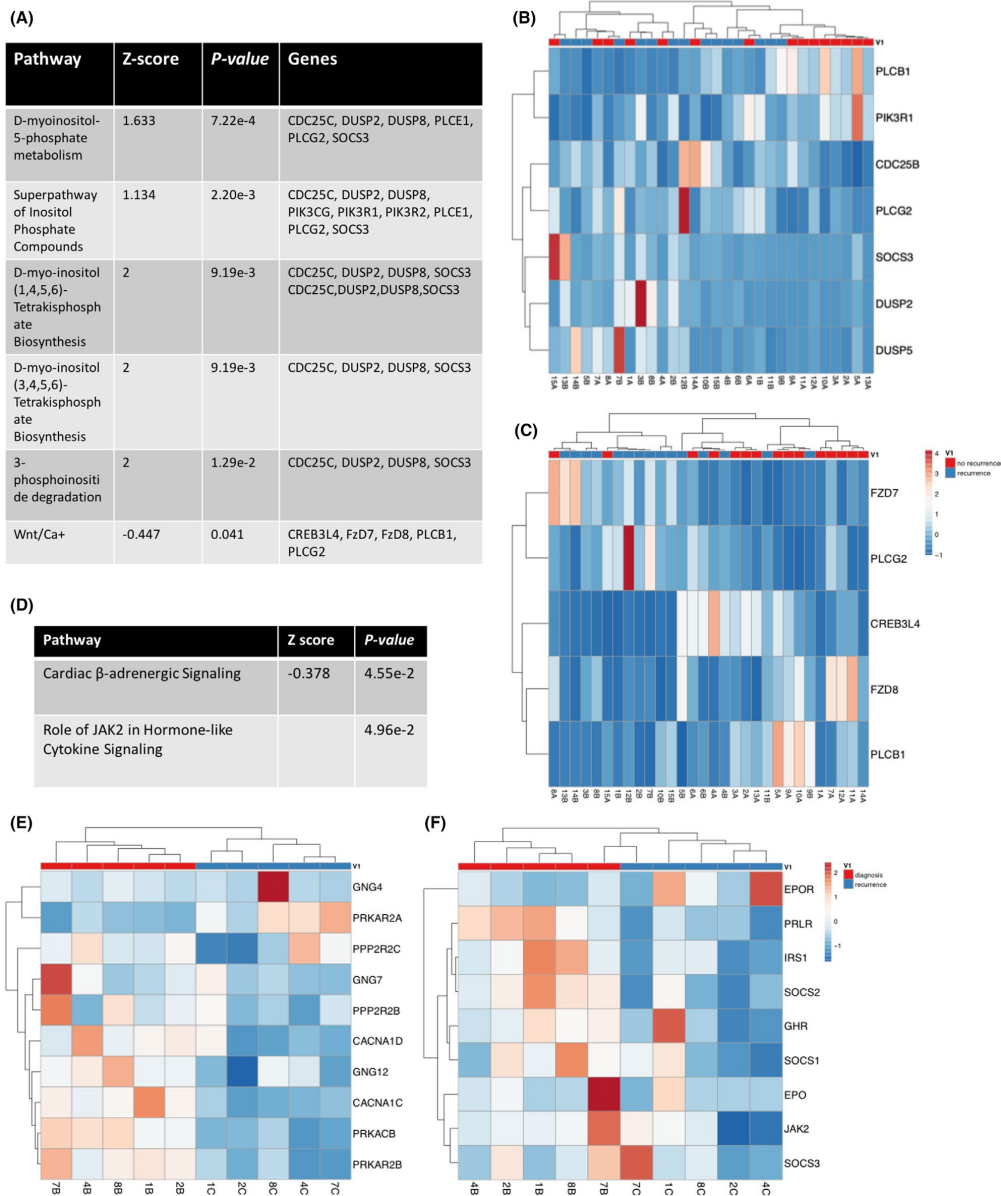
5 Ingenuity analysis identified multiple pathways significantly altered in H‐IR EMCA in relation to disease recurrence. (A) Table of the top significantly altered pathways when comparing primary pathology for patients with no disease recurrence versus patients with recurrence. Heatmaps showing differential expression of individual genes present in the (B) five metabolic pathway genes and (C) Wnt/Ca^+^ signaling pathways. (D) Table of top significantly altered pathways when comparing pathology for patient's primary versus recurrent tumor. Heatmaps showing differential expression of individual genes present the (E) cardiac β‐adrenergic signaling and (F) role of JAK2 in hormone‐like cytokine signaling pathways. H‐IR EMCA, high‐intermediate risk endometrial cancer

When comparing genetic pathways in the tumors from H‐IR EMCA patients with disease recurrence to patients with no disease recurrence, six pathways were found to be significantly altered. These included: (1) D‐myo‐inositol‐5‐phosphate metabolism (*p* = 0.0007; *Z* score = 1.633), (2) Superpathway of inositol phosphate compounds (*p* = 0.002; *Z* score = 1.134), (3 and 4) D‐myo‐inositol (1,4,5,6)‐tetrakisphosphate biosynthesis (*p* = 0.009; *Z* score = 2 and *p* = 0.009; *Z* score = 2), (5) 3‐phosphoinositide degradation (*p* = 0.01; *Z* score = 2), and (6) Wnt/Ca^+^ (*p* = 0.041; *Z* score = −0.447) (Figure 5A). However, the Wnt/Ca^+^ pathway was significantly downregulated in patients with disease recurrence versus patients with no disease recurrence. The genes in each of these pathways are listed with their corresponding heatmap (Figure 5B,C). When analyzing genetic pathways for the five patients with both primary and recurrent tumor pathology, two of the most significantly altered pathways included cardiac β‐adrenergic signaling (*Z* score = −0.378; *p* = 0.0455) and the role of JAK2 in hormone‐like cytokine signaling (*p* = 0.0496) (Figure 5D). All pathways were downregulated in the patient's recurrent versus initial tumors. Genes in each of these pathways are listed in their respective heatmaps (Figure 5E,F).

### 3.4 CADD analysis highlights a deleterious gene profile in H‐IR EMCA patients

**FIGURE 6:**
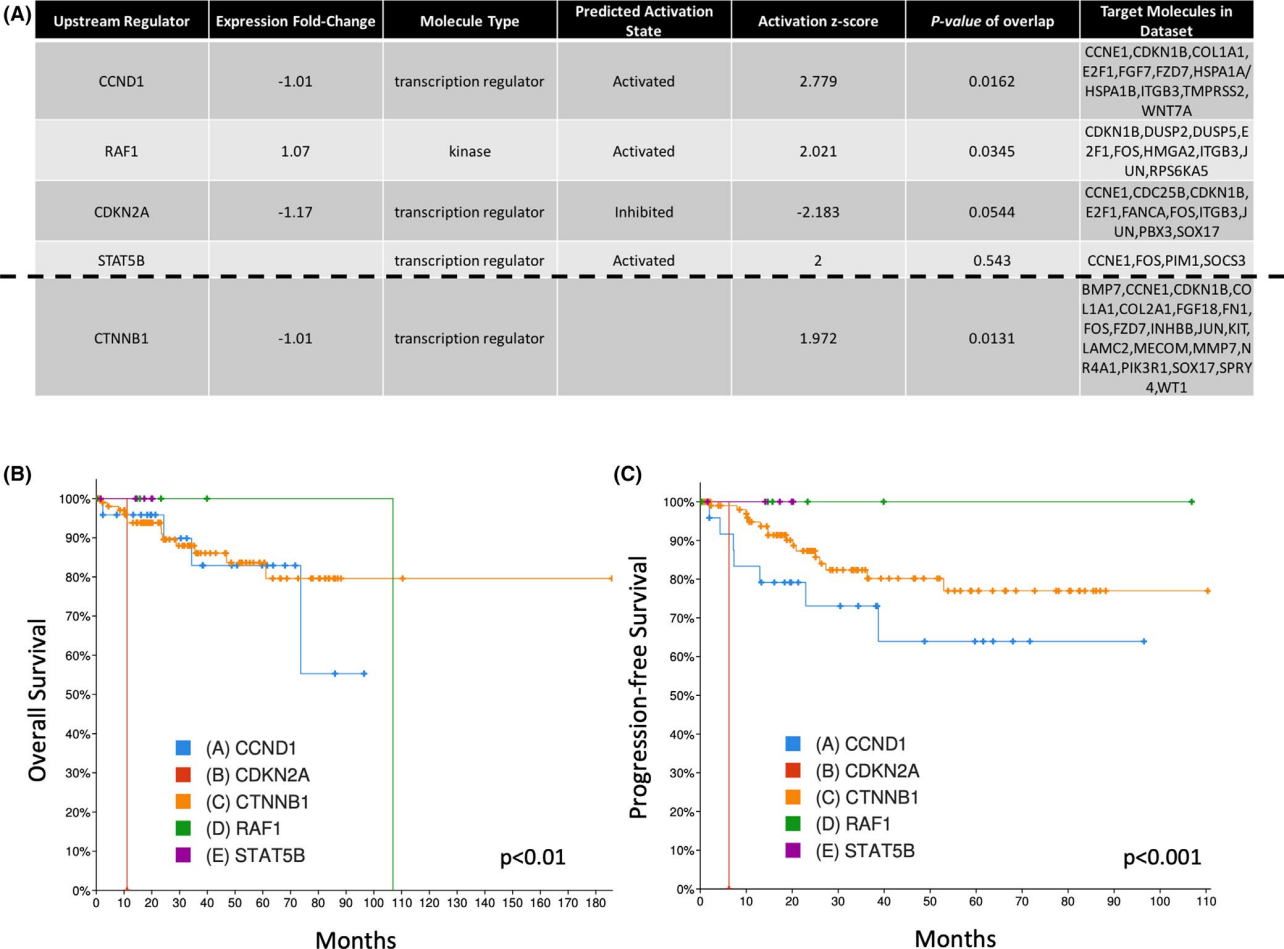
6 CADD highlights genes with likely deleterious mutations that affect survival in patients with H‐IR EMCA. (A) Table of upstream regulators with a CADD score >30 show multiple genes with likely deleterious alterations and their respective downstream molecular targets. Uterine Corpus Endometrial Carcinoma (TCGA, PanCancer Atlas) dataset (*n* = 529) from cBioPortal for Cancer Genomics illustrating the effects of gene mutations on (B) overall survival and (C) progression‐free survival. CADD, combined annotation‐dependent depletion; H‐IR EMCA, high‐intermediate risk endometrial cancer

When utilizing CADD analysis, we discovered that *SMARCA4*, *KDM5A*, *TET1*, *EPHB1*, *TGFBR2*, *CCND1*, *RAF1*, *CTNNB1*, *CDKN2A*, and *STAT5B* all have CADD scores >30. These results signify that these genetic mutations are likely deleterious, and account for some of the most deleterious mutations. Each of these genes identified from NGS have multiple downstream targets as shown in Figure 6A. The downstream targets were identified from the RNA sequencing gene list and were altered as a consequence of the DNA mutations. Of these genes, CCND1, RAF1, and STAT5B all had *Z*‐scores >2 (CTNNB1 *Z*‐score = 1.97), meaning they are significantly *activated*, while CDKN2A had a *Z*‐score <−2 indicating that it is significantly *inhibited* (Figure 6A). Figure 6B,C shows how these individual gene mutations affect patient OS and PFS, respectively, in TCGA_Endo dataset.

## 4 DISCUSSION

When H‐IR EMCA patients are grouped together based on modified GOG‐99 criteria, the recurrence risk is ~25%; however, we hypothesized that molecular data in addition to pathologic and clinical features could better stratify these patients and distinguish those with higher or lower risks of recurrence. Dou et al. performed proteomic analysis on EMCA tumors and were able to successful quantify protein, phosphorylation, and acetylation. That study provided function information through these assessments, then further characterized EMCA biology and highlighted new approaches to clinical management; however, it did not discuss recurrence.^18^ Adjuvant therapy has been shown to reduce the risk of H‐IR EMCA recurrence but does not improve the OS and is not without risks of treatment. Identifying molecular profiles to consistently predict the higher likelihood of recurrence could help clinicians better tailor their decision‐making regarding which H‐IR EMCA patients warrant the cost and potential toxicity of adjuvant treatment. This would facilitate more efficient use of healthcare resources while preventing lower risk patients from receiving unnecessary interventions.

This study investigated tumor molecular profiles from a full cohort (“Arend cohort”) of H‐IR EMCA patients who did not receive adjuvant therapy and their association with disease recurrence. DNA analyses revealed a strong correlation in the top 10 most frequently mutated genes in primary tumors from the Arend (30 patients) and TCGA (393 patients) cohorts, including PTEN, ARID1A, PIK3CA, PIK3R1, KMT2D, and CTNNB1. These data suggest that the Arend cohort, despite comprising a small number, is representative of the larger molecular landscape of EMCA patients analyzed in the TCGA cohort. A strength of our study is that all patients in the Arend cohort were universally observed and did not receive adjuvant therapy, eliminating this potential confounder; moving forward, it will be important to distinguish the different molecular responses between H‐IR EMCA patients who did and did not receive adjuvant therapy. Overall, the concordance between cohorts supports the utilization of our molecular findings for future studies investigating in clinically useful biomarkers.

When comparing gene mutations in individual patients’ primary and recurrent tumor tissue, DNA analyses found only a 30% overlap in gene mutations between the two groups. This suggests that the mutations that persisted are likely vital for tumorigenic function throughout the disease course. However, there was an increase in gene mutation percentage in an individual patient's recurrent tumor tissue compared to their primary tumor, suggesting that the recurrent tumor acquired additional mutations that could be driver mutations to developing recurrence and/or therapeutic resistance. Furthermore, DNA analyses found that three of five patients with primary and recurrent tumors developed new mutations in *TP53*, *LRP1B*, *CARD11*, *CCND3*, *GATA3*, and *PRDM3* in their recurrent tumors. We also identified 32 genes with enhanced gene expression in the patient's recurrent versus primary tumor, suggesting there are indeed molecular profiles associated with disease recurrence. Additionally, 47 genes were found to have decreased expression in the patient's recurrent versus primary tumor, suggesting that expression of these genes corresponds to a decreased risk of recurrence; thus, patients harboring this gene signature may not warrant the toxicity and expense of receiving adjuvant therapy. Of note, one of the genes in this signature, LEFTY2, is associated with stemness in ovarian cancer,^19^ a known contributor of chemoresistance.

RNA analyses revealed 14 genes that had enhanced expression in H‐IR EMCA patients with disease recurrence compared to patients without recurrence: components of this gene signature corresponded to decreased patient survival in the TCGA_Endo dataset. This suggests that increased expression of these genes corresponds to an increased recurrence risk; thus, patients harboring this gene signature may warrant the toxicity and expense of receiving adjuvant therapy. In addition, four genes (BAIAO3, MECOM, MAP3K13, and NOS) were found to have decreased expression in tumor from patients who experienced recurrence compared to those who did not. This suggests that expression of these genes corresponds to a decreased risk of recurrence; thus, patients harboring this gene signature may not warrant the toxicity and expense of receiving adjuvant therapy. As an example, *MECOM* expression has shown to be associated with therapeutic resistance in ovarian serous carcinoma.^20^ These findings support the hypothesis that molecular testing for RNA expression patterns, which include both upregulation and downregulation of specific genes could facilitate clinician decision‐making that limit unnecessary adjuvant radiation and/or chemotherapy.

Of note, there was an increased incidence of recurrence in Black compared to White patients in the Arend cohort (71% and 43%). The genes that were upregulated in the Black patient recurrent tumors were associated with increased morbidity. Racial and ethnic differences in endometrial cancer outcomes represent an active area of research: however, racial disparities must be recognized as multifactorial in nature, including socioeconomic factors, discordant receipt of appropriate treatment, and systemic racism as components that influence patient care and outcomes.^21^, ^22^ Our findings support potential biological differences between groups that may facilitate personalized treatment decisions; however, the complexity of racial disparities in endometrial cancer should not be oversimplified to a purely genetic etiology. With respect to racial differences, our findings support the need for further research and acknowledge the importance of an approach that incorporates the multitude of factors influencing health disparities prior to drawing conclusions. Within the context of these analyses, further avenues may include identifying factors that cause mutational pattern differences as seen in the Arend cohort.

Ingenuity pathway analyses revealed metabolic pathways significantly upregulated in the tumor from patients that recurred versus those that did not. Cancer stem cells (CSCs) have been shown to have increased metabolic pathway activation (CSCs),^23^ and these cells are known to enhance chemotherapeutic resistance that aids in tumor recurrence. During our CADD analysis, *CCND1*, *RAF1*, and *STAT5B* were found to be significantly activated, while *CDKN2A* was significantly inactivated in H‐IR EMCA patient tumors. Cyclin D1 (encoded by *CCND1*) enhances the aggressive nature of CSCs by promoting cellular epithelial to mesenchymal transition (EMT), migration, proliferation, and drug resistance.^24^ In addition, previous findings have highlighted *RAF1* as a top promoter of mesenchymal stem cell (MSC) function.^25^ MSCs have self‐renewing, secretory, and immunomodulatory (immune dampening) capabilities^25^ and are known to regulate CSCs through paracrine mechanisms.^26^ Stat5b (encoded by *STAT5B*) has been characterized to induce EMT and stem‐like properties via Jak2‐Stat5a/b signalingy.^27^ Thus, our data identified genes activated in the tumor from patients that recurred that are known regulators of tumor recurrence and therapeutic resistance in CSCs.

This study utilized NGS by CARIS and NanoString RNA analysis, which could both be available to physicians. These findings suggest that gene profiles could be utilized to predict which patients should receive adjuvant therapy, but perhaps even more important––the patients in which the toxicity and cost of adjuvant therapy are NOT warranted. No conclusions can be drawn on patient survival or clinical impact without further studies involving larger cohorts. Limiting overtreatment is of significant interest, as adjuvant therapy can cause significant short‐term and long‐term toxicities. The cost to patients and the healthcare system for overtreatment and related complications must also be considered. Currently, the algorithm to determine H‐IR patients and those within that group at highest risk leaves notable ambiguity. Our preliminary findings suggest that gene expression via a commercially available platform that can perform RNA analysis on archival FFPE tissue could eventually aid in this decision‐making process. Further investigation with an expanded dataset is warranted to better characterize and validate these results.

The major limitations of this study include small sample size and lack of diversity within samples. In conclusion, further research is needed to provide additional avenues to counsel patients and facilitate clinician decision‐making regarding adjuvant treatment in H‐IR EMCA patients.

## CONFLICT OF INTEREST

RCA has participated in Advisory Boards for Leap Therapeutics, AstraZeneca, GSK, Merck, VBL Therapeutics, and Caris Life Sciences. All other authors have no conflicts of interest. CAL has participated on Grant Funding boards for NIH, Advisory Boards for Eisai and GSK, and Contracted Research for Merck and AstraZeneca.

## ETHICS APPROVAL

Under an Institutional Review Board‐approved protocol at the University of Alabama at Birmingham (UAB), all patients with biopsy‐proven EMCA who underwent surgery between 2000 and 2010 at UAB and met criteria for H‐IR EMCA disease based on GOG‐99 criteria (although outer 1/3 was replaced by outer 1/2) were reviewed.

## Supporting information

Fig S1‐S3

## Data Availability

The data that support the findings of this study are available upon request from the corresponding author.
