# Supplementary material for: Identifying a molecular profile to predict the risk of recurrence in high‐intermediate risk endometrial cancer
Source: Cancer Med. 2021 Nov 2;10(22):8238–50. doi: 10.1002/cam4.4247 (PMC8607249; doi:10.1002/cam4.4247)
Supplement: Supplementary file 1 — Fig S1‐S3 [file CAM4-10-8238-s001.pdf]

A.

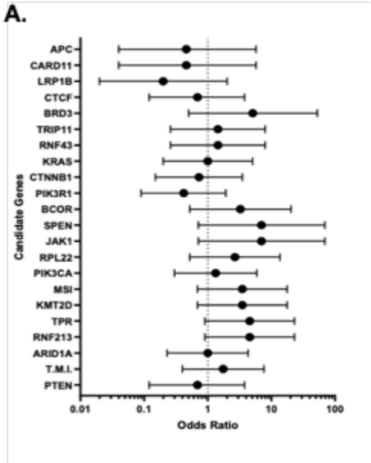

	Odds Ratio	95% CI (lower)	95% CI (upper)
JAK1	7	0.71	69.49
SPEN	7	0.71	69.49
BRD3	5.09	0.5	52.59
RNF213	4.57	0.9	23.14
TPR	4.57	0.9	23.14
KMT2D	3.5	0.69	17.71
MSI	3.5	0.69	17.71
BCOR	3.25	0.52	20.37
RPL22	2.67	0.52	13.66
T.M.I.	1.75	0.4	7.66
RNF43	1.45	0.26	8.01
TRIP11	1.45	0.26	8.01
PIK3CA	1.33	0.3	5.91
ARID1A	1	0.23	4.31
KRAS	1	0.2	5.04
CTNNB1	0.73	0.15	3.49
PTEN	0.69	0.12	3.79
CTCF	0.69	0.12	3.79
CARD11	0.46	0.04	5.75
APC	0.46	0.04	5.75
PIK3R1	0.42	0.09	1.92
LRP1B	0.2	0.02	2.02

Survival analysis (JAK1, SPEN, BRD3, RNF213, TPR)

B. C.

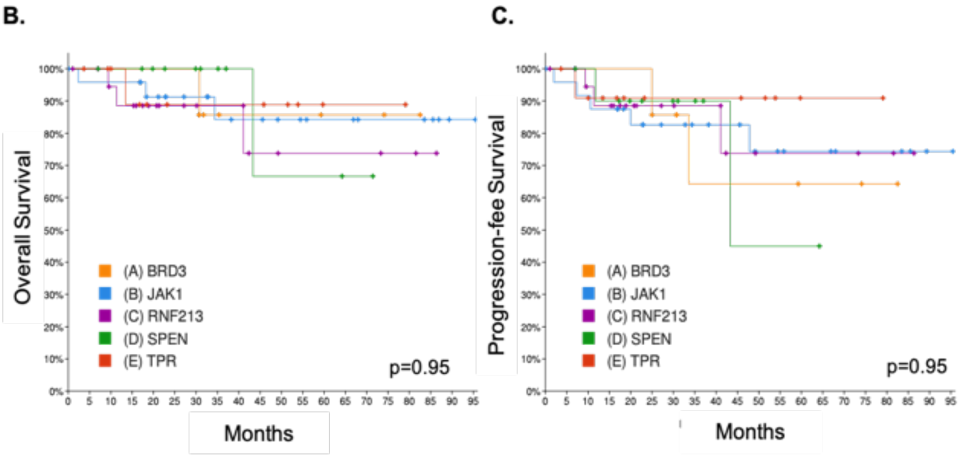

Figure S1. Odds ratio for common genes nonrecurrent vs recurrent H-IR EMCA patient tumors. Tumor DNA was isolated and followed by NextSeq. All variants were detected with >99% confidence based on allele frequency and average coverage of >500 and an analytic sensitivity of 5%. Genetic variants identified were interpreted by board-certified molecular geneticists and categorized based on American College of Medical Genetics (ACMG) standards. **(A)** Odds ratios and 95% confidence intervals (95% CI) were calculated then verified using an online odds ratio calculator tool (https://select-statistics.co.uk/calculators/confidence-interval-calculator-odds-ratio/). **(B)** Primary tumors in both the recurrent and non-recurrent H-IR EMCA patients had expression of JAK1, SPEN, BRD3, RNF213, and TPR (odds ratios > 1); combined expression of this gene profile correlated with a significant decrease in patient OS.

A.

	Gene	Fold-Change (Recurrent to Non-recurrent) African American population
Top 15 genes **increased**	SOST	18.80
	HMGA2	11.23
	DKK4	10.67
	IL10	6.22
	FST	4.12
	FGF3	3.69
	ETV4	3.64
	FN1	3.36
	HOXA9	3.16
	MET	2.63
	CCNE1	2.62
	SPP1	2.59
	GNG4	2.57
	ITGA8	2,56
Top 15 genes **decreased**	IL1R2	-1.51
	MAPK8IP2	-1.52
	GNG7	-1.53
	BIRC3	-1.54
	GADD45B	-1.55
	KLF4	-1.56
	DKK1	-1.56
	CACNA2D3	-1.58
	CACNA1H	-1.61
	AMH	-1.64
	FGF10	-1.64
	FOXL2	-1.69
	IL8	-1.71
	MM9	-1.75
	IL19	-1.81

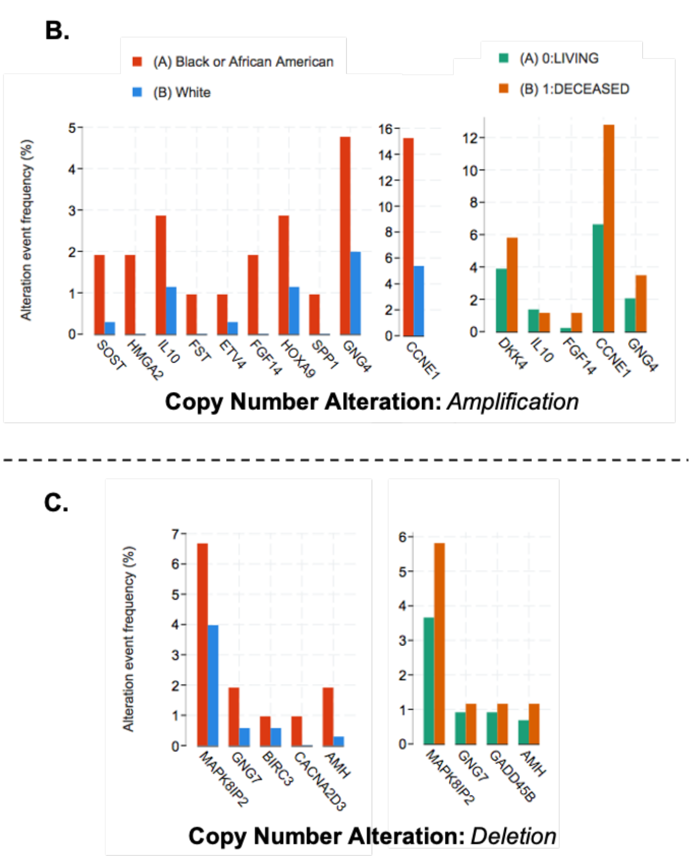

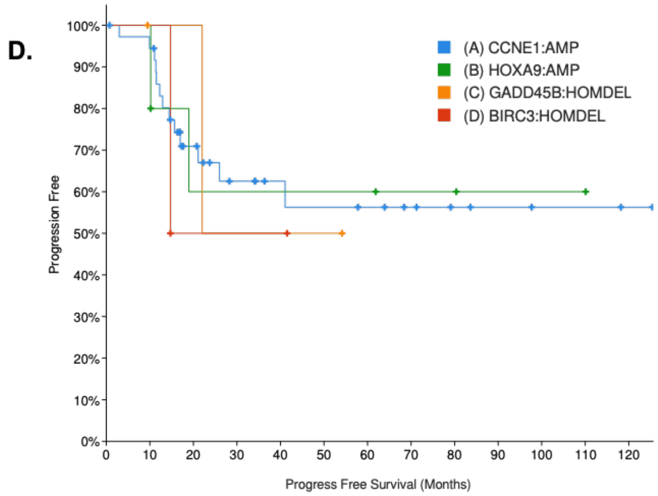

Figure S2. African American recurrent vs. White recurrent gene expression profiles. (A) Expression fold-change of the top 15 increased (top – white) and top 15 decreased (bottom – grey) genes in African American recurrent (n=5) vs. White recurrent (n=10) H-IR EMCA patient populations. Uterine Corpus Endometrial Carcinoma (TCGA, PanCancer Atlas) dataset (n=529) from cBioPortal for Cancer Genomics illustrating the frequency of copy-number alterations (CNAs) of **(B)** amplified (AMP) or **(C)** homozygous deleted genes between in African American (red) and White (blue) patient populations, and their association with patient morbidity (green/orange). **(D)** The effects of AMP or HOMDEL genes on progression-free survival (PFS) in Uterine Corpus Endometrial Carcinoma (TCGA, PanCancer Atlas) dataset.

A.

	Gene	Fold-Change (Recurrent to Non-recurrent) African American population
Top 15 genes **increased**	DKK4	204.1177358
	HMGA2	34.71036415
	SOST	18.79963636
	ZIC2	13.84944984
	NR4A1	12.73055556
	NR4A3	10.86914661
	BMP7	9.966801256
	SFRP2	9.553090909
	NKD1	9.043598139
	FN1	8.364171324
	IL10	7.469090909
	FGF19	7.455636364
	BMP4	7.44438861
	SPP1	7.360872687
	FGF3	7.050545455
Top 15 genes **decreased**	AKT1	-1.564318
	FGF12	-1.5649487
	THEM4	-1.5819235
	SOX17	-1.6276556
	PLCB1	-1.6412897
	PLCB4	-1.6543518
	MYB	-1.6893392
	FZD8	-1.7006766
	NOS3	-1.7057976
	IDH1	-1.7090183
	MAP3K13	-1.7132306
	IL5RA	-1.716948
	BAIAP3	-1.7382937
	SIX1	-1.7822172
	GPC4	-1.7951976

B.

C.

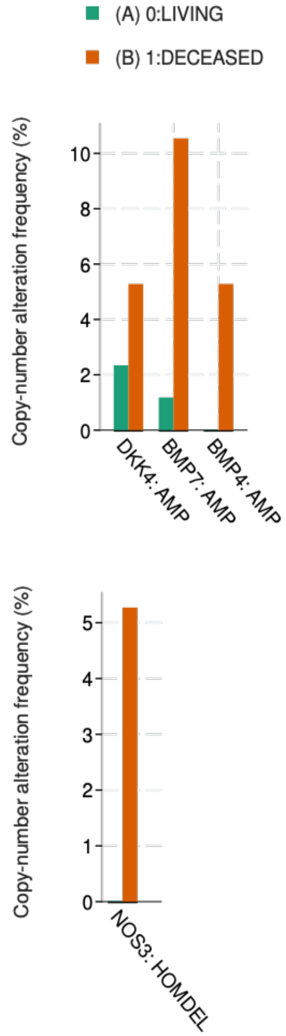

Figure S3. African American recurrent vs. non-recurrent tumor gene expression profiles. **(A)** Expression fold-change of the top 15 increased (top – white) and top 15 decreased (bottom – grey) genes in African American recurrent (n=5) vs. non-recurrent (n=2) H-IR EMCA patient populations. Uterine Corpus Endometrial Carcinoma (TCGA, PanCancer Atlas) dataset (n=529) from cBioPortal for Cancer Genomics illustrating the association of copy-number alteration (CNA) of **(B)** amplified (AMP) or **(C)** homozygous deletion (HOMDEL) of genes and patient morbidity.
